# Middle Ear Salivary Gland Choristoma: A Case Report

**Published:** 2017-01-02

**Authors:** Katayoun Ziari, Kamyab Alizadeh

**Affiliations:** *Dept. of Pathology, AJA University of Medical Sciences, Tehran, Iran*

**Keywords:** Salivary Gland, Choristoma, Middle Ear

## Abstract

Salivary gland choristoma of the middle ear cavity is a very rare condition. These lesions are a result of a defective embryonic development and their adjacent structures may be associated with abnormalities. Here we report a case of salivary gland choristoma of the middle ear who presented to Be’sat Hospital, Tehran, Iran in 2015 with unilateral conductive hearing loss. There are 41 case reports in English and non-English literature from 1961. Taylor and Martin reported the first case of middle ear salivary choristoma.

## Introduction

Choristoma is not a tumor, but it is a histologically normal mature tissue located in an ectopic position (1). The presence of a normal salivary gland tissue in a different anatomical position than salivary gland is defined as salivary gland choristoma (2). 

Salivary gland choristoma of the middle ear cavity was first reported by Taylor and Martin in 1961, and since then only 41 cases have been reported (3). The most cases of choristoma have been reported with other abnormalities of the ossicular chain and facial nerve (4, 5). 

Here, a typical case of salivary gland choristoma of the middle ear is described who presented with conductive hearing loss in his left ear developed during the patient’s childhood.

## Case report

**Fig. 1:**
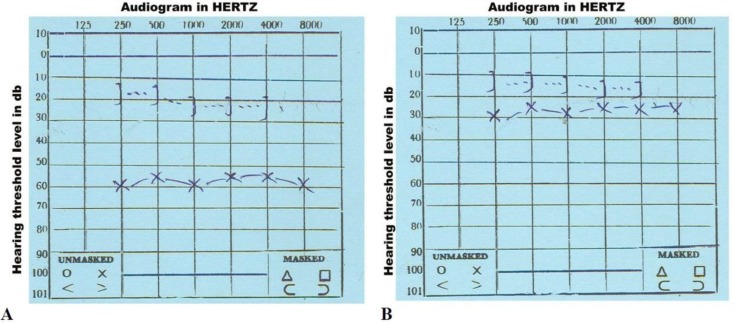
Left ear audiogram of patient: (A) preoperation (B) postoperation

A 39-yr-old man with a hearing loss that had persisted since childhood in his left ear and an intermittent otorrhea and otalgia was presented to Be’sat Hospital, Tehran, Iran in 2015. The previous episode of otorrhea and otalgia from childhood treated with antibiotics findings from his physical examination showed no external deformities of his auricle and external ear canal. Otoscopic examination revealed opacity at the lower part of his left tympanic membrane. Pure tone audiometry results were normal in his right ear, but in the left ear demonstrated 56-db conductive hearing loss with normal bone conduction (Fig. 1). The mastoid aeration of the left temporal bone was lost, and a soft tissue mass filling the hypotympanum level of left middle ear was identified, detected in the temporal bone CT scan of the patient. Other clinical and paraclinical examination results such as CBC, urine analysis, liver function test, chest x-ray, and electrocardiography were within normal limit. Informed consent was taken from the patient.

**Fig. 2:**
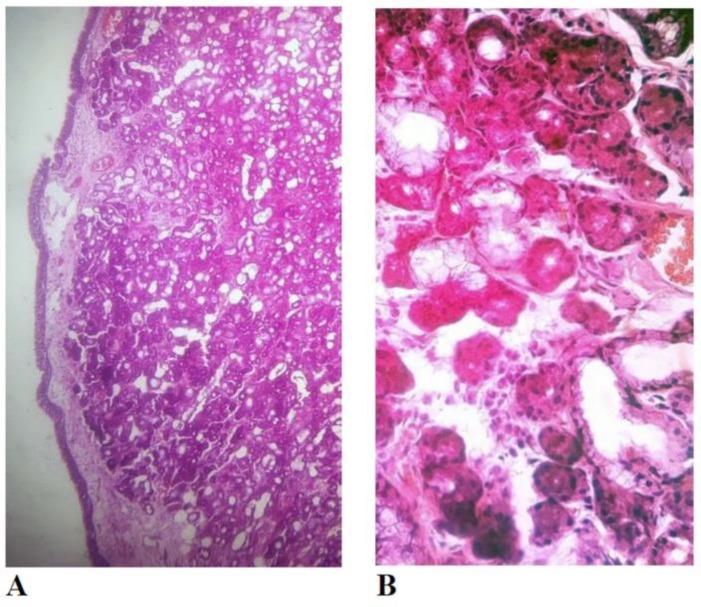
A) Low power picture shows a mass composed of mucous and serous glands resembling normal salivary tissue covered with the middle-ear mucosa (H&E; x 40). (B) High power picture exhibits detailed glands (H&E; x 400

Exploratory tympanotomy of the left ear was performed under general anesthesia. A 1 0.7 cm yellowish mass adherent to the mucosa of the promontorium was found. No evidence of cholesteatoma was observed. The malleus and the incus were intact, but the stapes had been eroded by the mass. The mass was easily dissected. The respiratory epithelium covering seromucinous glands were found in histopathologic examination of the removed mass (Fig. 2). Pure tone audiometry two months after the operation showed the patient’s hearing was almost normal and the patient was uneventful.

## Discussion

The middle ear salivary gland choristoma is a rare condition. About 41 cases (including the present case) have been reported in the literature (6, 7). Although it is unclear the precise pathogenesis of this disease, its occurrence is before the fourth month of gestation (4). However, it is associated with the ectopic expansion of the remnant parotid epithelium or pharyngeal endoderm (3). Salivary gland choristoma, hearing loss, ossicular chain abnormalities, facial nerve anomalies, and branchial arch anomalies are components of a syndrome (8). 

This lesion may accompany by other branchial arch anomalies (9). The distribution age of patients in various reports was between 9 months and 52 yr (10) with a ratio of two women to one man (3). The clinical feature of this disease is occurrence of salivary gland tissue in the middle ear that usually described as a unilateral lesion (96.8%), and the most frequently affected side was left (61.3%) (3, 10). Bilateral involvement is reported in postmortem evaluation of a patient (11).

Our patient was a 39-yr-old man with clinical features including unilaterality and left sided occurrence. This is in agreement with other studies (3, 10, 11). Hearing loss (usually a moderate to severe conductive or mixed types) is the most presenting symptom (93.6%) diagnosed during infancy or childhood (10). The present case was also admitted with unilateral hearing loss that is compatible with other literature (3, 6, 10, 12). Moreover, this patient had a recurrent episode of otorrhea and otalgia. Low otoscopic visualization of a mass in the tympanic cavity (37%) and high prevalence of conductive hearing loss among patients in the first decade of life lead to misdiagnosis of the condition (6).

Most injuries associated with middle ear salivary gland choristoma are due to the sole presence and action of the choristomatous mass and not resulting from embryological failure or abnormal development of the first and second branchial arches (6). Facial weakness is rare, despite high incidence of facial canal dehiscence and adherence to the mass (3, 6, 10).

In our case, involvement of the facial nerve or abnormalities in the course of the facial nerve was not observed. Any histopathological investigation can establish the definite diagnosis of salivary gland choristoma (2, 6). This lesion is required to be differentiated from other middle ear mass includes congenital cholesteatoma, dermoid cyst, teratoma, glomus tympanicum, granuloma, neuroma, or glioma which are presenting with unilateral hearing loss without perforation of the tympanic membrane (1, 3, 10, 13). 

Treatment is determined by the size and location of the tumor (10). Conservative management, biopsy, and serial examination have been recommended when the mass is intimately associated with facial nerve (6, 10, 14). In small and pedunculated tumors, total excision is recommended (14). Salivary tumor including benign and malignant have been reported in middle ear (15-17), this lesion possibly originated from choristomatous salivary tissue, thus if possible, total excise is recommended (14). In our case, the mass was easily dissected because there was no evidence of mass involvement of the facial nerve. 

## Conclusion

Salivary gland choristoma is an unusual condition and. It should be diagnosed and differentiated from other lesions of the middle ear that lead to conductive hearing loss. However, in pedunculated and small tumors, total excision has been recommended without causing permanent damage to the facial nerve, because several cases with malignant neoplasm arisen from choristoma have been reported.

## Conflict of Interests

The authors declare that there is no Conflict of Interests. 
